# Anthelmintic activity of European fern extracts against *Haemonchus contortus*

**DOI:** 10.1186/s13567-023-01192-8

**Published:** 2023-07-13

**Authors:** Antonio Pavičić, Markéta Zajíčková, Michaela Šadibolová, Gabriela Svobodová, Petra Matoušková, Barbora Szotáková, Lenka Langhansová, Petr Maršík, Lenka Skálová

**Affiliations:** 1 Department of Biochemical Sciences, Faculty of Pharmacy, Charles University, Heyrovského 1203, 50005 Hradec Králové, Czech Republic; 2 Institute of Experimental Botany, Czech Academy of Sciences, Rozvojová 263, 16502 Prague, Czech Republic

**Keywords:** Natural anthelmintics, medicinal plants, nematodes, ATP-assay, *Athyrium*, *Dryopteris*

## Abstract

Most drugs used in the treatment of helminthiasis in humans and animals have lost their efficacy due to the development of drug-resistance in helminths. Moreover, since anthelmintics, like many pharmaceuticals, are now recognized as hazardous contaminants of the environment, returning to medicinal plants and their products represents an environmentally friendly way to treat helminthiasis. The goal of the present study was to test the anthelminthic activity of methanol extracts of eight selected European ferns from the genera *Dryopteris*, *Athyrium* and *Blechnum* against the nematode *Haemonchus contortus*, a widespread parasite of small ruminants. Eggs and adults of *H. contortus* drug-susceptible strain ISE and drug-resistant strain WR were isolated from experimentally infected sheep. The efficacy of fern extracts was assayed using egg hatch test and adults viability test based on ATP-level measurement. Among the ferns tested, only *Dryopteris aemula* extract (0.2 mg/mL) inhibited eggs hatching by 25% in comparison to control. *Athyrium distentifolium*, *Dryopteris aemula* and *Dryopteris cambrensis* were effective against *H. contortus* adults. In concentration 0.1 mg/mL, *A. distentifolium*, *D. aemula*, *D. cambrensis* significantly decreased the viability of females from ISE and WR strains to 36.2%, 51.9%, 32.9% and to 35.3%, 27.0%, 23.3%, respectively in comparison to untreated controls. None of the extracts exhibited toxicity in precise cut slices from ovine liver. Polyphenol’s analysis identified quercetin, kaempferol, luteolin, 3-hydroxybenzoic acid, caffeic acid, coumaric acid and protocatechuic acid as the major components of these anthelmintically active ferns.

## Introduction

Parasitic nematodes cause serious livestock diseases worldwide, afflicting hundreds of millions of food-producing animals. The anthelmintics; benzimidazoles, imidazothiazoles, tetrahydropyrimidines and macrocyclic lactones used for decades in the prevention and treatment of helminths’ infections have lost their effectiveness due to the development of resistance. Such resistance has been reported on all continents except Antarctica [1]. Due to the socio-economic impact of nematode infections of livestock and human populations as well as the worldwide spread of anthelmintic resistance, there is an urgent need to discover and develop new drugs for the sustained and effective control of nematodes [2].

New anthelmintics development, involves four different approaches, each of them with certain advantages and disadvantages [3]. The first, classical approach is the synthesis of new chemical entities active against parasitic nematodes [4]. The synthesis of derivatives of known drugs with an approved usage represents another approach [5]. The third approach of drug repurposing is based on finding new anthelmintically active substances among drugs approved for other diseases [6]. These three approaches have in common the fact that they use pharmaceuticals that are now considered to be hazardous environmental contaminants. In particular, the use of these substances in large quantities in the treatment of livestock puts a heavy burden on the environment and poses high risks to ecosystems [1, 7–10]. On the other hand, returning to medicinal plants and their products, which is the fourth alternative approach to control helminthiasis, represents an environmentally friendly way.

Despite the efficacy of medicinal plants in the treatment of parasitic diseases which has been known and used for centuries, the use of plant-based prophylaxis and therapies to treat parasitosis has been stifled by the expansion and availability of synthetic drugs [11]. Nevertheless, plants remain a major source for the development and discovery of new therapeutics, with countless diverse active compounds with anthelmintic activities identified from plant sources [12]. Moreover, increasing public and professional interest in the ecological impact of pharmaceuticals has stimulated a revival in the search for and use of medicinal plants in helminthiasis treatment [13–15]. In addition to other higher plants, ferns (*Pteridophytes*) have emerged as an interesting group of vascular plants which have attracted attention in terms of their potential use in medicine [16–18]. Accordingly, the purpose of our project is to identify a fern that shows anthelmintic effects and could be added (in a proper form) to livestock feed to replace (at least partly) or supplement synthetic anthelmintics.

In presented study, the anthelminthic activity of methanol extracts from eight selected European ferns from the genera *Dryopteris*, *Athyrium* and *Blechnum* was tested. The nematode *Haemonchus contortus*, was used as a model species. *H. contortus* is a hematophagous gastrointestinal parasite of small ruminants which is extremely detrimental to animal health and productivity on a global scale. Moreover, *H. contortus* is able to effectively defend itself against anthelmintic drugs, with field populations of this species now showing resistance to all major anthelmintic drug classes [19]. Therefore, new therapy against *H. contortus* is extremely needed. The effect of the fern extracts was tested in *H. contortus* eggs and adults of both drug-sensitive (ISE, Inbred-Susceptible-Edinburgh, MHco3) and drug-resistant strain (WR, White River, MHco4) of the nematode. In addition, the potential toxicity of these extracts was assayed in ovine liver, as sheep represent a common host of *H. contortus*. In the three fern species determined as active, the content of the main polyphenols was analyzed.

## Materials and methods

### Chemicals and reagents

The Pierce^™^ BCA Protein Assay Kit and Williams’ Medium E-GlutaMAX (32551) was purchased from Thermo Fisher Scientific (Prague, Czech Republic). The standards of polyphenols and all other chemicals were purchased from Sigma-Aldrich (Prague, Czech Republic).

### Ferns and their extraction

Eight European fern species (*Athyrium distentifolium*, *Athyrium filix-femina*, *Blechnum spicant*, *Dryopteris aemula*, *Dryopteris borreri*, *Dryopteris cambrensis*, *Dryopteris dilatata*, *Dryopteris remota*) were used in this study. Plant material was collected in 2019 and 2020 from the Garden center of Jakub Krulich, Prague, Czech Republic, Garden center Franc, Kamenné Žehrovice, Czech Republic, or the private fern collection of RNDr. Libor Ekrt, Ph.D. (University of South Bohemia) in Telč, Czech Republic (Table 1). Each sample represented approximately 50 g fresh weight of mature leaves of one fern species collected out of three individual plants at minimum. Plant material collection and extraction methods have been described in Langhansova et al. [20]. Briefly, the fern leaves were freeze-dried, homogenized using a mortar, pestle, and liquid nitrogen. The tissue was extracted overnight in a ratio of 1 g of dry mass and 20 mL of MeOH p.a. The extract was separated from the plant tissue by centrifugation at 1730 × *g*, 15 min (Centrifuge Hettich Universal 32R, Tuttlingen, Germany) at room temperature, with the supernatant filtered through a 30 mm PVDF 0, 45 µm membrane filter (ProFill, Fisher Scientific, Pardubice, Czech Republic). The extracts were reduced in a vacuum in a rotary evaporator and dried completely under a nitrogen flow. The obtained dry extracts were redissolved in dimethyl sulfoxide (DMSO) at a concentration of 100 mg/mL, with the DMSO aliquots maintained at −80 °C until use. The extract yield was on average 27% of the dry mass.

**Table 1:** **List of tested fern species.**

	*Species*	Common name	*Family*	Source of plant material
F1	*Athyrium distentifolium*	Alpine lady fern	*Aspleniaceae*	Ekrt
F2	*Athyrium filix-femina*	Lady fern	*Aspleniaceae*	Krulich
F3	*Blechnum spicant*	Hard fern, Deer fern	*Blechnaceae*	Franc
F4	*Dryopteris aemula*	Hay scented buckler fern	*Dryopteridaceae*	Ekrt
F5	*Dryopteris borreri*	Borrer’s male-fern	*Dryopteridaceae*	Ekrt
F6	*Dryopteris cambrensis*	Narrow male-fern	*Dryopteridaceae*	Ekrt
F7	*Dryopteris dilatata*	Broad buckler fern	*Dryopteridaceae*	Franc
F8	*Dryopteris remota*	Scaly buckler-fern	*Dryopteridaceae*	Ekrt

### Infecting sheep

In one experiment, two lambs were infected with ISE strain and two lambs with WR strain. Three independent experiments were performed. All experimental procedures were approved by the Ethics Committee of the Ministry of Education, Youth and Sports (Protocol MSMT-25908/2019) and performed in accordance with Czech Act No 246/1992 Coll. on the Protection of Animals against Cruelty.

Six-month-old lambs were firstly dewormed by albendazole (5 mg/kg), after which the feces were microscopically examined for the absence of parasites. Three weeks later, when the lambs were free of parasites, they were orally infected with L3 larvae of *Haemonchus contortus* ISE and WR strain. Prior to infection, the stock L3 were incubated overnight (25 °C) in sieve with pore diameter 20–25 µM to remove dead larvae. Only the living L3 could pass through the sieve, and those were used for infection. The infection dose was dependent on the weight of the animal and ranged between 6000 and 8000 L3.

### Isolation of *H. contortus* eggs

The eggs were isolated from ovine feces 4 weeks after infection. Around the anus of each lamb, plastic bags were fastened to the hair using clips, into which feces were continuously excreted. When some bag was full, the new bag was used. The feces were collected within 1 day and one night. As the temperature in sheep stable was approx. 10 °C, the hatching of eggs during feces collecting was limited and the amounts of larvae was negligible. The feces in cooled boxes were transported in laboratory, then pooled together and mashed. The fecal mass was passed through 3 sieves with a decreasing pore diameter (250, 100, and 25 µm) to remove coarse particles. The eggs were captured in the last sieve together with the mud and transferred to the 50 µL falcon tubes and centrifuged for 3 min (481 × *g*). The supernatant was then replaced by sucrose flotation solution (FS, saturated sucrose solution with density 1.27 g/cm^3^), mixed and centrifuged for 3 min (188 × *g*). The eggs floating on the top were collected into new 15 mL falcons with FS and centrifugation was repeated. The eggs were then cleaned by repeated centrifugation in tap water (481 × *g*). Freshly isolated eggs were used immediately for the egg hatch test.

### Egg hatch test

Firstly, the eggs of *H. contortus* were diluted to concentration 25 eggs/100 µL. The stock solutions of fern extracts (or albendazole as a positive control) were diluted with DMSO, and 1 µL of these solutions (or 1 µL of DMSO for the negative control) of each concentration were pipetted into one well of 96-well plates in 8 biological replicates. Then 199 µL of egg suspension was added to each well, and the eggs were incubated at 27 °C for 48 h. The final tested concentrations of fern extracts were 12.5; 25; 50; 100 and 200 µg/mL; final concentration of DMSO in all samples and controls was 0.5% and each well contained approximately 50 eggs. The incubation was halted by adding 5 µL of concentrated Lugol’s iodine solution. The proportion of unhatched eggs and larvae was counted under the microscope and compared to the control. Two independent experiments in eight biological replicates were performed.

### Isolation of *H. contortus* adults

The adults of *H. contortus* were isolated by the agar method as described previously [21]. In brief, the sheep were euthanized 6 weeks post infection, and their abomasa were removed. The adult nematodes were released together with the abomasa content into a beaker filled with physiological solution (PHS, 0.9% NaCl, 37 °C). When the nematodes sedimented to the bottom of the beaker, the extra PHS was sucked out, and the remaining fluid containing adult nematodes was mixed with agar in a proportion of 1:1. This agar mixture was then poured in a thin layer over gauzes. Once the mixture solidified, the gauzes were placed into aquariums filled with temperate PHS and incubated (37 °C) until the alive adults extracted themselves out of the agar and sedimented to the bottom of the beaker. The adults were then collected and cleaned, then manually separated according to gender and used immediately for the experiments.

### Viability assay on *H. contortus* adults

The viability testing was based on the measurement of remaining ATP in the adult worms after 48 h of incubation with increasing concentrations of the respective fern extract being tested. Levamisole (as a positive control) was used to check the correctness of the test performance.

The fern extracts and levamisole were pre-dissolved in DMSO, then dissolved in supplemented RMPI-1640 medium. The medium was supplemented according to [21] with glucose (0.8%), amphotericin B (0.25 µg/mL), penicillin (10 U/mL), streptomycin (10 µg/mL) and HEPES (10 mM; (N-[2-hydroxyethyl] piperazine-N′-[4-butanesulfonic acid] buffer, pH 6.8). The assay was performed according to [22, 23] with the following adjustments: Firstly, 8 male or 4 female adults were placed into one well of 24-well plate with 1 mL RMPI-1640 media containing 1.0; 10; 50; and 100 µg/mL fern extract (or only 0.1% DMSO for the control samples) and incubated in a humid atmosphere at 37 °C for 48 h. After incubation, the worms from each well were washed in PBS and placed into a 2 mL plastic tube containing 100 µL of SONOP (sonification solution, 70% ethanol with 2 mM EDTA (ethylenediaminetetraacetic acid); pH 10.9) and immediately frozen in dry ice. The samples were stored at −80 °C until measurement.

To measure ATP level, the samples were firstly homogenized in 700 µL of cooled Tris/EDTA buffer (100 mM Tris–HCl; 2 mM EDTA; pH 7.8) in FastPrep-24 5G homogenizer (MP Biomedicals, Irvine, CA, USA) for 20 s, following which 700 µL of fresh Tris/EDTA buffer was added and homogenization was repeated. The samples were centrifuged (Eppendorf, 12 000 × *g*, 10 min, 4 °C) and ATP was measured in 5 µL of supernatant pipetted from each sample onto one well of a 96-well black plate. The volume of each well was topped up to 50 µL with Tris/EDTA buffer. 50 µL of Luciferase was added right before the measurement, and the value of luminescence was measured within 5 min (Spark Control Tecan, v. 2.2). The amount of ATP was calculated from a calibration curve and normalized to the µg of total protein measured in the supernatant according to the manufacturer’s protocol (Pierce™ BCA Protein Assay Kit). The values of ATP in the control samples were considered as 100% viability. Reagents from ATP Bioluminescence assay kit CLS II (Roche, Mannheim, Germany) were used for the experiment.

### Hepatotoxicity testing

The liver lobes removed immediately after the sheep were euthanized were placed into Euro-Collins’ solution and transported to the laboratory within 20 min. Precision-cut liver slices (PCLS) were used to determine the potential hepatotoxic effect of each of the selected fern extracts to ovine liver. The PCLS preparation and the measurement of ATP level (as a viability marker) were performed according to [24]. In brief, 8 mm wide and 150–170 µm thick liver slices were preincubated for 1 h in 1 mL of Williams’ E Medium (with L-glutamine, Invitrogen, Paisley, UK) supplemented with glucose (final concentration 36 mM) and 50 μg/mL gentamycin at 37 °C in an atmosphere of 85% O_2_ and 5% CO_2_. Then PCLS were placed into 1.3 mL of fresh media (temperature 37 °C) containing fern extracts at the concentration 100 µg/mL (or 10% DMSO, which kills all PCLS, as a positive control and 0.1% DMSO as a negative control) and incubated for 24 h in the same conditions. After incubation the PCLS were collected separately, washed in PBS, and placed into 150 µL of SONOP and immediately frozen on dry ice and stored at −80 °C until measurement.

Prior to ATP measurement, the samples were homogenized in 1 mL of SONOP (FastPrep-24 5G homogenizer) and centrifuged (centrifuge Eppendorf, 12 000 × *g*, 5 min, 4 °C). ATP was determined in 5 µL of supernatant as described above (paragraph 2.7.). The total protein content used for correction was measured by BCA assay (Pierce^™^ BCA Protein Assay Kit) in the remaining sample pellet after SONOP evaporation. The pellet was then dissolved in 200 µL of 5 M NaOH (60 min, 37 °C), and diluted with redistilled water to 1 mL. The calibration plot was also prepared in 1 M NaOH.

### Analysis of polyphenol content in fern extracts

The extracts were dissolved in 100% MeOH and maintained for 24 h at −18 °C to precipitate protein, centrifuged (15 000 rpm for 10 min at 4 °C), with the supernatant collected into vials. The purified extracts were then analyzed using an UHPLC/MS-HRAM system consisting of high-resolution accurate-mass (HRAM) Q-TOF spectrometer Impact II (Bruker Daltonik, Germany) coupled with Ultimate 3000 chromatograph (Thermo Fisher Scientific, USA). Chromatographic separation was performed using an Acclaim RSLC 120 C18 column (2.2 µm, 2.1 × 100 mm, Thermo Fisher Scientific, USA) and gradient elution with mobile phases 0.2% formic acid (A) and 100% methanol (B). The gradient started at 2% B (0–2 min), and then was ramped from 2 to 100% B (2–15 min), maintained at 100% B (15–20 min), returned to starting conditions 2% B (20–21 min) and equilibrated at 2% B (21–26 min). The flow rate was 250 µL/min, and column temperature was maintained at 40 °C. Detection was performed in positive mode using an ESI ion source with mass resolution 60 000. Injection volume was 5 µL. The measured compounds were identified through a comparison of their exact mass and retention time with commercial standards (Sigma-Aldrich, Czech Republic). MS data were acquired using oTof Control 4.0 and HyStar 3.2 software, with qualitative and quantitative analysis carried out by DataAnalysis 4.3 and TASQ 4 software, respectively (all Bruker Daltonics, Germany).

### Statistical analysis

For the statistical analyses GraphPad Prism 9.1.2. software was used. Two-way ANOVA with Dunnett’s multiple comparisons test was used to process the data.

## Results

### Effect of fern extracts on eggs hatching

In the ISE strain, only the F4 extract and only at the highest concentration tested (200 µg/mL) decreased eggs hatching significantly by approximately 25%. In lower concentrations, none of the tested fern extracts showed a significant effect on *H. contortus* ISE eggs hatching. The results are expressed as a percentage of hatched eggs compared to control and summarized in Table 2. In the WR strain, no effect of any fern extract on eggs hatching was observed (data not shown). The IC_50_ for positive control albendazole was 1.9 and 4.4 µM in ISE and WR strain, respectively.

**Table 2:** **The effect of the fern extracts on hatching of *H. contortus*** **eggs (ISE strain).**

	Control	12.5 µg/mL	Percentage of hatched eggs [%]		100 µg/mL	200 µg/mL
			25 µg/mL	50 µg/mL		
F1—*Athyrium distentifolium*	100.0 ± 4.79	100.6 ± 0.01	98.8 ± 14.2	93.6 ± 1.11	99.7 ± 3.90	100.8 ± 11.0
F2—*Athyrium filix-femina*	100.0 ± 4.79	98.6 ± 0.55	94.3 ± 1.85	90.5 ± 1.10	90.4 ± 8.97	85.6 ± 4.56
F3—*Blechnum spicant*	100.0 ± 4.79	101.4 ± 1.71	101.6 ± 0.70	96.1 ± 5.81	99.5 ± 2.04	100.0 ± 0.57
F4—*Dryopteris aemula*	100.0 ± 4.79	96.6 ± 2.98	97.6 ± 4.14	99.5 ± 0.30	99.8 ± 0.08	76.0 ± 27.0^a^
F5—*Dryopteris borreri*	100.0 ± 4.79	102.6 ± 4.37	91.0 ± 2.53	94.8 ± 2.12	93.9 ± 2.35	97.1 ± 2.86
F6—*Dryopteris cambrensis*	100.0 ± 4.79	98.1 ± 4.62	100.6 ± 1.45	98.1 ± 0.89	101.9 ± 3.46	96.6 ± 0.66
F7—*Dryopteris dilatata*	100.0 ± 4.79	113.8 ± 14.6	109.4 ± 8.60	105.0 ± 11.7	109.2 ± 3.99	103.7 ± 11.5
F8—*Dryopteris remota*	100.0 ± 4.79	96.5 ± 1.24	97.9 ± 0.79	96.5 ± 5.85	96.5 ± 6.06	96.6 ± 5.26

The data are presented as percentage of hatched eggs (as the means ± SEM). The data were obtained from two independent experiments in eight biological replicates.

^a^Significant decrease (*p* ≤ 0.05) comparing to the control sample, two-way ANOVA.

### Effects of fern extracts on the ATP level in H. contortus adults

**Figure 1:**
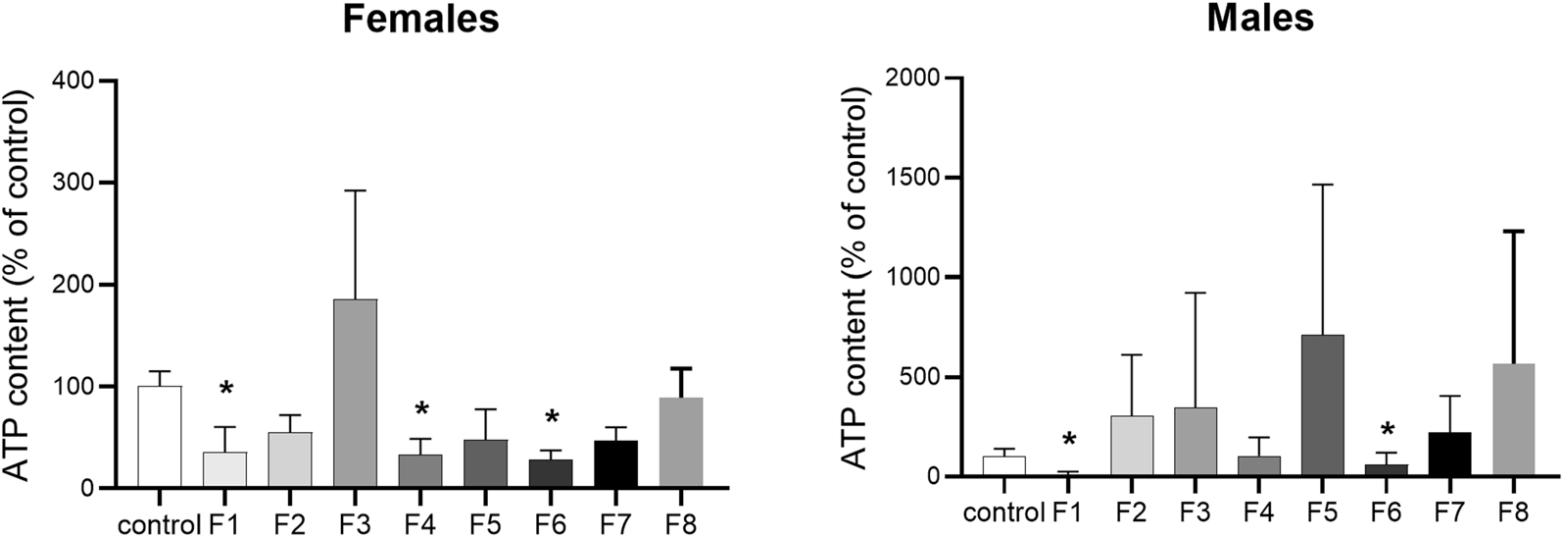
**The effect of 100 µg/mL fern extracts on viability of *H. contortus* ISE strain adults.** The results are presented as means ± SEM. The data were obtained from one experiment in eight biological replicates. For statistical analysis, two-way ANOVA with Dunnett’s multiple comparisons test was used, * *p* < 0.05.

Eight fern extracts (in concentration 100 µg/mL) were tested in adults of *H. contortus* ISE strain. Only extracts of F1, F4, and F6 significantly diminished the level of ATP in *H. contortus* adults, F1 and F6 in males and F1, F4, and F6 in females (Figure 1).

**Figure 2:**
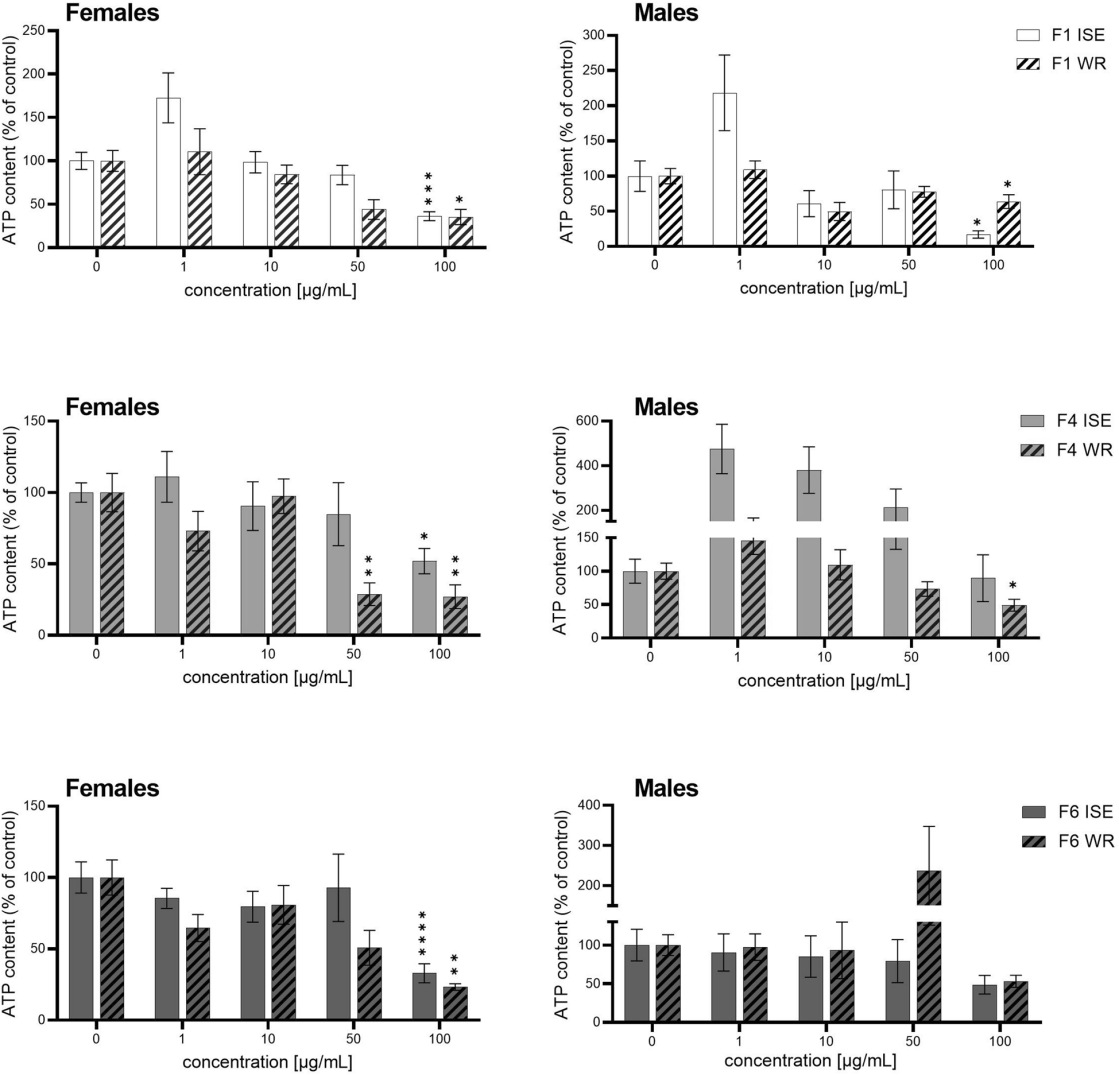
**Comparison of the effect of selected fern extracts on the viability of *H. contortus* adults in the ISE and WR strain.** The viability of *H. contortus* adults in cultivation medium without fern extracts (zero concentration) represent the controls. The data were obtained from three independent experiments, each in 4–6 biological replicates. The results are presented as means ± SEM. For statistical analysis, two-way ANOVA with Dunnett’s multiple comparisons test was used, * *p* < 0.05; ** *p* < 0.01; *** *p* < 0.001; **** *p* < 0.0001.

Based on these results, ferns F1, F4, and F6 were selected for further testing at various concentrations (1, 10, 50, and 100 µg/mL) with adults of both ISE and WR strains. The results are presented in Table 3 (ISE strain) and Table 4 (WR strain). In concentration 100 µg/mL, F1, F4 and F6 significantly decreased the ATP level in females from ISE strain to 36.2%, 51.9%, 32.9% in comparison to untreated controls. In the males of ISE strain, the effect of F4 and F6 was less pronounced than in the females. On the other hand, F1 was more active in the males of ISE, decreasing ATP level to 17.1%. In the females of WR strain, F1, F4 and F6 (in concentration 100 µg/mL) significantly decreased the ATP level to 35.3%, 27.0%, 23.3%, respectively, in comparison to untreated controls. Moreover, F4 was effective also in concentration 50 µg/mL decreasing the ATP level to 28.8%. In the males of WR strain, the F1 and F4 (in concentration 100 µg/mL) significantly decreased ATP level to 63.8 and 49.0% in comparison to control. The comparison of the ferns effect in adults of ISE and WR strains is presented in Figure 2. The IC_50_ for positive control levamisole was 6.3 µM and 36.5 µM in males of ISE and WR strain, respectively.

**Table 3:** **ATP content of *H. contortus*** **adults of ISE strain after 48 h incubation of males and females with ferns extract of** ***Athyrium distentifolium*, *Dryopteris aemula* and *Dryopteris cambrensis***.

	ATP content (%)									
	Females					Males				
	Control	1 µg/mL	10 µg/mL	50 µg/mL	100 µg/mL	Control	1 µg/mL	10 µg/mL	50 µg/mL	100 µg/mL
*A. distentifolium*	100.0 ± 10.0	172.6 ± 28.7	98.5 ± 12.3	83.7 ± 11.1	36.2 ± 5.1	100.0 ± 21.5	218.3 ± 53.8	60.9 ± 18.5	80.5 ± 26.8	17.1 ± 5.1
*D. aemula*	100.0 ± 6.8	111.0 ± 17.8	90.5 ± 17.1	84.9 ± 22.0	52.0 ± 8.9	100.0 ± 18.1	475.1 ± 110.6	379.9 ± 104.2	214.0 ± 81.3	89.5 ± 35.1
*D. cambrensis*	100.0 ± 10.9	85.4 ± 7.1	79.5 ± 10.8	92.8 ± 23.6	32.9 ± 6.7	100.0 ± 20.5	90.6 ± 24.1	85.3 ± 26.8	79.3 ± 27.9	48.7 ± 12.1

The data are presented as percentage of ATP content in comparison to control (means ± SEM). The data were obtained from three independent experiments, each in 4–6 biological replicates.

**Table 4:** **ATP content of *H. contortus* adults of WR strain after 48 h incubation of males and females with ferns extract of *Athyrium distentifolium*, *Dryopteris aemula* and *Dryopteris cambrensis***.

	ATP content (%)									
	Females					Males				
	Control	1 µg/mL	10 µg/mL	50 µg/mL	100 µg/mL	Control	1 µg/mL	10 µg/mL	50 µg/mL	100 µg/mL
*A. distentifolium*	100.0 ± 12.0	110.5 ± 26.4	84.3 ± 10.7	44.0 ± 11.4	35.3 ± 8.8	100.0 ± 10.8	109.2 ± 12.6	49.7 ± 12.8	77.8 ± 7.6	63.8 ± 9.7
*D. aemula*	100.0 ± 13.4	73.0 ± 13.8	97.5 ± 12.2	28.8 ± 7.9	27.0 ± 8.3	100.0 ± 12.0	145.4 ± 20.5	109.4 ± 22.7	73.4 ± 10.9	49.0 ± 9.0
*D. cambrensis*	100.0 ± 12.4	64.5 ± 9.5	80.9 ± 13.6	50.7 ± 12.2	23.3 ± 2.3	100.0 ± 13.5	97.5 ± 17.2	93.2 ± 36.6	236.6 ± 110.7	53.2 ± 7.8

The data are presented as percentage of ATP content in comparison to control (means ± SEM). The data were obtained from three independent experiments, each in 4–6 biological replicates.

### The test of potential hepatotoxicity

The three anthelmintically active ferns F1, F4, and F6 were tested for potential hepatotoxic effects in the ovine liver. The viability of precision-cut liver slices (PCLS) after 24 h incubation with the tested fern extracts in concentration 100 µg/mL was assayed. The amount of ATP per mg of protein in PCLS exposed to the fern extracts and in the control PCLS was measured and compared. The ATP level (expressed as percentage of control) of PCLS exposed to F1, F4, and F6 extracts was 107.8 ± 3.58%; 132.6 ± 40.86%; and 125.3 ± 8.3%, respectively. Therefore, none of the ferns tested (in concentration 100 µg/mL) showed a significant hepatotoxic effect on ovine liver.

### The polyphenols in fern extracts

The content of polyphenols in the extracts of F1, F4, and F6 was analyzed, with the major components quantified using commercially available standards. The results showed significant differences (both qualitative and quantitative) in polyphenols content among ferns tested. Only quercetin was present in a relatively high amount in all the extracts. Taxifolin was abundant in F4, although in F1 it was detected in a much lower amount, and not present at all in F6. On the other hand, luteolin, a major component of F6, was detected only in traces in F4, and it was not found in F1. Coumaric acid and caffeic acid were detected only in F4, vitexin only in F1. A much greater amount of 3-hydroxybenzoic acid was present in F6 than in F1 and F4. The results are presented in Table 5 and the content of particular polyphenols is expressed in µg per gram of dry extract.

**Table 5:** **The quantification of polyphenols in fern methanolic extracts.**

	*Athyrium distentifolium*	*Dryopteris aemula*	*Dryopteris cambrensis*
	µg/g of dry extract		
3-Hydroxybenzoic acid	14.0	44.9	186
Caffeic acid		21.0	
Chlorogenic acid	0.08	2.25	0.17
Ferulic acid			
Kaempferol	93.1	15.9	8.15
Luteolin		7.35	136
Luteolin glucoside			
p-Coumaric acid		31.6	
Protokatechuic acid	12.4	22.3	22.1
Quercetin	479	247	172
Rutin			0.50
Taxifolin	47.9	267	
Vitexin	13.3		

## Discussion

In previous study, some European fern species from genus *Dryopteris*, *Athyrium* and *Blechnum* were shown to exhibit antioxidative, anti-inflammatory and anticancer activity [20]. In our present study, the anthelmintic activity of these ferns was tested against parasitic nematodes, with *Haemonchus contortus* (family *Trichostrongylidae*), a hematophagous gastrointestinal parasite of small ruminants, chosen as the model for this purpose. The drug- resistance in *H. contortus* is widespread in field populations showing resistance to all available anthelmintics [25]. When excrements of sheep and goats from 34 farms in mid-Atlantic U.S. regions were analyzed, *H. contortus* was the most common parasite found in feces, resistant to benzimidazoles almost in 100% of farms [26]. For these reasons, there is a great need to find and determine new active compounds which will be also effective against resistant strains of *H. contortus*.

Among eight fern extracts tested, the extracts of *Athyrium distentifolium* (F1), *Dryopteris aemula* (F4), and *Dryopteris cambrensis* (F6) were effective against *H. contortus* adults in the concentration 100 µg/mL. Higher concentrations could not be tested due to limited solubility and the risk of undesired interference with bioluminescent detection, thus IC_50_ values could not be calculated. Interestingly, in some cases lower concentrations of fern extract increased the ATP level. This might mean that lower concentrations of extracts did not kill the nematodes but evoked stress, which is manifested by increased ATP production. Nevertheless, it must be emphasized that extracts of *A. distentifolium* and *D. cambrensis* were effective not only in the drug-susceptible strain ISE, but also in the drug-resistant strain WR. In addition, the results are valuable also because adults of *H. contortus* were used for testing and not more easily obtainable larvae. Adults represent the parasitic developmental stage that a potential drug should target. If the tested substance is effective on adults ex vivo, there is a greater chance that it could also be effective in vivo*.* The measurement of ATP level as a viability marker has been optimized for use in *H. contortus* recently and represent the relatively simple biochemical method for anthelmintic efficacy testing [23].

*D. aemula* (at the highest concentration tested) diminished the hatching of eggs. This indicates that this extract might prevent the larval development of *H. contortus* in the feces of treated animals, which could partially reduce the risk of reinfection. The inhibitory effect on eggs’ development is an advantage of this extract which many widely-used anthelmintics (ivermectin, levamisole, monepantel) do not show. In addition, none of the three extracts exhibited toxicity in ovine liver. This fact is very important for approval of planned in vivo study.

The anthelmintic effect of some ferns has been reported previously. *Blechnum orientale* extract (5 mg/mL) showed mild anthelmintic activity against isolated *Gastrothylax crumenifer* adult trematodes [17]. *D. filix-mas* (2 mg/mL) extract had in vitro moderate nematocidal activity against the infective third-stage larvae of *Trichostrongylus colubriformis* [27] Comparing these data with our results, the extracts from *A. distentifolium*, *D. aemula* and *D. cambrensis* seem to be promising, as they were effective in much lower concentrations (0.1 mg/mL).

With aim to know the composition of these anthelmintically active extracts, the content of polyphenols was analyzed. The results showed significant qualitative and quantitative inter-species differences. The major components were quercetin, 3-hydroxybenzoic acid, kaempferol, and protocatechuic acid (detected in all three extracts), luteolin and taxifolin (detected in two extracts), caffeic acid and p-coumaric acid (detected only in *D. aemula*) and vitexin (only in *A. distentifolium*). In the present study, the anthelmintic effect of pure individual polyphenols was not tested, as several previous studies have already addressed this. When many polyphenols were tested in larvae of *H. contortus*, taxifolin proved to be ineffective, whereas quercetin and luteolin were highly effective [28]. Kaempferol and quercetin were the major component of extracts from *Pithecellobium dulce* (Robx.) with ovicidal activity against *H. contortus* [29]. The relatively high level of 3- hydroxybenzoic acid was found in the fruit of the Surinam cherry (*Eugenia uniflora L.*), which exhibits anthelmintic effects in the free-living nematode *Caenorhabditis elegans* [30]. The activity of caffeic acid against *H. contortus* as well as other nematodes has been reported [31, 32].

Although several polyphenols exhibit anthelmintic activity, they could act synergistically or antagonistically in helminths when used together as a complex mixture of compounds (as whole plant or plant extracts). From practical point of view, the effect of these “natural mixtures” is important because for farmers it would be cheaper to add fresh or dry plants (e.g., ferns) into fodder for livestock than to use an anthelmintic drug (even one based on pure polyphenols). Similarly, the anthelmintic activity of many medicinal plants and natural products has been tested in vivo, and in some cases has been proven effective [33, 34]. Based on our ex vivo results, the ferns of *A. distentifolium*, *D. aemula*, and *D. cambrensis* species would also merit testing in vivo. Only in vivo study in sheep infected with *H. contortus* could show the anthelmintic efficacy of these ferns. For these experiments, we might make following estimation: since the extracts were effective beginning at concentration 0.1 mg/mL, 1 g of fresh fern leaves prepared for the preparation of 67 mg of extract, the volume of ovine plasma is 3–4 L, and the bioavailability of polyphenols is low (below 1–10%), one sheep should be fed with at least 100 g of fresh fern leaves (or 25 g of dry fern leaves) to achieve the anthelmintic effect in sheep infected with *H. contortus.*

The extract from European ferns *Athyrium distentifolium*, *Dryopteris aemula*, and *Dryopteris cambrensis* exhibited significant anthelmintic activity in concentrations 0.1 mg/mL against the adults of parasitic nematodes *H. contortus* ex vivo.

## References

[1] Ahuir-Baraja AE, Cibot F, Llobat L, Garijo MM (2021). Anthelmintic resistance: is a solution possible?. Exp Parasitol.

[2] Lanusse C, Canton C, Virkel G, Alvarez L, Costa L, Lifschitz A (2018). Strategies to optimize the efficacy of anthelmintic drugs in ruminants. Trends Parasitol.

[3] Zajíčková M, Nguyen LT, Skálová L, Raisová Stuchlíková L, Matoušková P (2020). Anthelmintics in the future: current trends in the discovery and development of new drugs against gastrointestinal nematodes. Drug Discov Today.

[4] Geary TG (2016). *Haemonchus contortus*: applications in drug discovery*. Haemonchus Contortus* and Haemonchosis—past, present and future trends. edited by gasser RB Von Samson-Himmelstjerna G. Adv Parasitol.

[5] Panic G, Duthaler U, Speich B, Keiser J (2014). Repurposing drugs for the treatment and control of helminth infections. Int J Parasitol Drugs Drug Resist.

[6] Weeks JC, Roberts WM, Leasure C, Suzuki BM, Robinson KJ, Currey H, Wangchuk P, Eichenberger RM, Saxton AD, Bird TD, Kraemer BC, Loukas A, Hawdon JM, Caffrey CR, Liachko NF (2018). Sertraline, paroxetine, and chlorpromazine are rapidly acting anthelmintic drugs capable of clinical repurposing. Sci Rep.

[7] Bilkova Z, Mala J, Hrich K (2019). Fate and behaviour of veterinary sulphonamides under denitrifying conditions. Sci Total Environ.

[8] Kim B, Ji K, Kim C, Kang H, Lee S, Kwon B, Kho Y, Park K, Kim K, Choi K (2019). Pharmaceutical residues in streams near concentrated animal feeding operations of Korea—occurrences and associated ecological risks. Sci Total Environ.

[9] Vokral I, Sadibolova M, Podlipna R, Lamka J, Prchal L, Sobotova D, Lokvencova K, Szotakova B, Skalova L (2019). Ivermectin environmental impact: excretion profile in sheep and phytotoxic effect in *Sinapis alba*. Ecotoxicol Environ Saf.

[10] Widiyanti PM, Sudarwanto MB, Sudarnika E, Widiastuti R (2019). The use of enrofloxacin antibiotic as a veterinary drug and its residual hazards on public health. Wartazoa.

[11] Geary TG, Chibale K, Abegaz B, Andrae-Marobela K, Ubalijoro E (2012). A new approach for anthelmintic discovery for humans. Trends Parasitol.

[12] Mukherjee N, Mukherjee S, Saini P, Roy P, Babu SPS (2016). Phenolics and terpenoids; the promising new search for anthelmintics: a critical review. Mini Rev Med Chem.

[13] Fayaz MR, Abbas RZ, Abbas A, Khan MK, Raza MA, Israr M, Khan JA, Mahmood MS, Saleemi MK, Rehman TU, Zaman MA, Sindhu ZUD (2019). Potential of botanical driven essential oils against *Haemonchus contortus* in small ruminants. Bol Latinoam Caribe Plantas Med Aromat.

[14] Santos FO, Cerqueira APM, Branco A, Batatinha MJM, Botura MB (2019). Anthelmintic activity of plants against gastrointestinal nematodes of goats: a review. Parasitology.

[15] Miro MV, Luque S, Cardozo P, Lloberas M, Sousa DM, Soares AMS, Costa LM, Virkei GL, Lifschitz AL (2020). Plant-derived compounds as a tool for the control of gastrointestinal nematodes: modulation of abamectin pharmacological action by carvone. Front Vet Sci.

[16] Das K, Rekha R, Ibrahim MA, Ahmed SY, Dang R (2017). Effect of demographic location on *Phlebodium decumanum* (Willd.) J. Sm. for its phytoconstituents and establishment of antioxidant and novel anthelmintic activity of its aqueous and methanolic leaf extracts. Ann Phytomedicine Int J.

[17] Devi RK, Vasantha S, Panneerselvam A, Rajesh NV, Jeyathilakan N (2016). Phytochemical constituents and in vitro trematocidal activity of Blechnum orientale Linn. against *Gastrothylax crumenifer*. Ann Phytomedicine Int J.

[18] Devi RK, Vasantha S, Panneerselvam A, Rajesh NV, Jeyathilakan N, Venkataramanan R (2017). *Gastrothylax crumenifer*: ultrastructure and histopathology study of in vitro trematodicidal effect of *Microlepia speluncae* (L.) Moore. J Appl Anim Res.

[19] Arsenopoulos KV, Fthenakis GC, Katsarou EI, Papadopoulos E (2021). Haemonchosis: a challenging parasitic infection of sheep and goats. Animals.

[20] Langhansova L, Pumprova K, Haisel D, Ekrt L, Pavicic A, Zajíčková M, Vanek T, Dvorakova M (2021). European ferns as rich sources of antioxidants in the human diet. Food Chem.

[21] van Wyk JA, Gerber HM, Groeneveld HT (1980). A technique for the recovery of nematodes from ruminants by migration from gastro-intestinal ingesta gelled in agar: large-scale application. Onderstepoort J Vet Res.

[22] Zajíčková M, Prchal L, Navrátilová M, Vodvárková N, Matoušková P, Vokřál I, Nguyen LT, Skálová L (2021). Sertraline as a new potential anthelmintic against Haemonchus contortus: toxicity, efficacy, and biotransformation. Vet Res.

[23] Nguyen LT, Zajíčková M, Mašátová E, Matoušková P, Skálová L (2021). The ATP bioluminescence assay: a new application and optimization for viability testing in the parasitic nematode *Haemonchus contortus*. Vet Res.

[24] Zárybnický T, Matoušková P, Lancošová B, Šubrt Z, Skálová L, Boušová I (2018). Inter-individual variability in acute toxicity of R-pulegone and R-menthofuran in human liver slices and their influence on miRNA expression changes in comparison to acetaminophen. Int J Mol Sci.

[25] Kotze AC, Prichard RK, Gasser RB, Von Samson-Himmelstjerna G (2016). Anthelmintic resistance in *Haemonchus contortus:* history, mechanisms and diagnosis. *Haemonchus Contortus* and *Haemonchosis—*past, present and future trends.

[26] Crook EK, O'Brien DJ, Howell SB, Storey BE, Whitley NC, Burke JM, Kaplan RM (2016). Prevalence of anthelmintic resistance on sheep and goat farms in the midAtlantic region and comparison of in vivo and in vitro detection methods. Small Rumin Res.

[27] Urban J, Kokoska L, Langrova I, Matejkova J (2008). In vitro anthelmintic effects of medicinal plants used in Czech Republic. Pharm Biol.

[28] Klongsiriwet C, Quijada J, Williams AR, Mueller-Harvey I, Williamson EM, Hoste H (2015). Synergistic inhibition of *Haemonchus contortus* exsheathment by flavonoid monomers and condensed tannins. Int J Parasitol Drugs Drug Resist.

[29] Olmedo-Juarez A, Jimenez-Chino AL, Bugarin A, Zamilpa A, Mendoza-de Gives P, Villa-Mancera A, Lopez-Arellano ME, Olivares-Perez J, Delgado-Nunez EJ, Gonzalez-Cortazar M (2022). Phenolic acids and flavonoids from *Pithecellobium dulce* (Robx.) benth leaves exhibit ovicidal activity against *Haemonchus contortus*. Plants.

[30] Rashmi HB, Negi PS (2022). Phytochemical constituents and anthelmintic potential of Surinam cherry (*Eugenia uniflora* L.) at different fruit developmental stages. South Afr J Botany.

[31] Escareno-Diaz S, Alonso-Diaz MA, de Gives PM, Castillo-Gallegos E, von Sonde FE (2019). Anthelmintic-like activity of polyphenolic compounds and their interactions against the cattle nematode *Cooperia punctata*. Vet Parasitol.

[32] Lima CS, Pereira MH, Gainza YA, Hoste H, Regasini LO, Chagas ACD (2021). Anthelmintic effect of *Pterogyne nitens* (Fabaceae) on eggs and larvae of *Haemonchus contortus*: analyses of structure-activity relationships based on phenolic compounds. Ind Crop Product.

[33] Adak M, Kumar P (2022). Herbal anthelmintic agents: a narrative review. J Trad Chine Med.

[34] Hoste H, Meza-Ocampos G, Marchand S, Sotiraki S, Sarasti K, Blomstrand BM, Williams AR, Thamsborg SM, Athanasiadou S, Enemark HL, Acosta JFT, Mancilla-Montelongo G, Castro CS, Costa LM, Louvandini H, Sousa DM, Salminen JP, Karonen M, Engstrom M, Charlier J, Niderkorn V, Morgan ER (2022). Use of agro-industrial by-products containing tannins for the integrated control of gastrointestinal nematodes in ruminants. Parasite.

